# From Suppressor T Cells to Regulatory T Cells: How the Journey that Began with the Discovery of the Toxic Effects of TCDD Led to Better Understanding of the Role of AhR in Immunoregulation

**DOI:** 10.3390/ijms21217849

**Published:** 2020-10-22

**Authors:** Narendra Prasad Singh, Mitzi Nagarkatti, Prakash Nagarkatti

**Affiliations:** Department of Pathology, Microbiology and Immunology, School of Medicine, University of South Carolina, Columbia, SC 29208, USA; narendra.singh@uscmed.sc.edu (N.P.S.); Mitzi.Nagarkatti@uscmed.sc.edu (M.N.)

**Keywords:** aryl hydrocarbon receptor (AhR), AhR Ligands, dioxin (TCDD), T regulatory cells (Tregs), Th17 cells (Th17), immunosuppression, inflammation, epigenetic pathways

## Abstract

Aryl hydrocarbon receptor (AhR) was identified in the early 1970s as a receptor for the ubiquitous environmental contaminant 2,3,7,8-tetrachlorodibenzo-p-dioxin (TCDD, dioxin), which is a member of halogenated aromatic hydrocarbons (HAHs). TCDD was found to be highly toxic to the immune system, causing thymic involution and suppression of a variety of T and B cell responses. The fact that environmental chemicals cause immunosuppression led to the emergence of a new field, immunotoxicology. While studies carried out in early 1980s demonstrated that TCDD induces suppressor T cells that attenuate the immune response to antigens, further studies on these cells were abandoned due to a lack of specific markers to identify such cells. Thus, it was not until 2001 when FoxP3 was identified as a master regulator of Regulatory T cells (Tregs) that the effect of AhR activation on immunoregulation was rekindled. The more recent research on AhR has led to the emergence of AhR as not only an environmental sensor but also as a key regulator of immune response, especially the differentiation of Tregs vs. Th17 cells, by a variety of endogenous, microbial, dietary, and environmental ligands. This review not only discusses how the role of AhR emerged from it being an environmental sensor to become a key immunoregulator, but also confers the identification of new AhR ligands, which are providing novel insights into the mechanisms of Treg vs. Th17 differentiation. Lastly, we discuss how AhR ligands can trigger epigenetic pathways, which may provide new opportunities to regulate inflammation and treat autoimmune diseases.

## 1. Introduction

Aryl hydrocarbon receptor (AhR) belongs to the family of basic helix–loop–helix transcription factors [1]. It was first discovered in the early 1970s for its ability to bind 2,3,7,8-Tetrachlorodibenzo-*p*-dioxin (TCDD) with high affinity [2] and induce a xenobiotic-metabolizing enzyme known as aryl hydrocarbon hydroxylase (AHH). TCDD, also known as dioxin, is an environmental contaminant that binds to AhR with high affinity. In the early 1970s, toxicologists also became aware of the fact that environmental contaminants can suppress the immune response leading to the emergence of a new field, immunotoxicology. In one of the early studies published in 1973, Vos et al., demonstrated that TCDD suppressed humoral and cell-mediated immunity [3]. These studies led to additional research all over the world exploring the effect of environmental pollutants and chemicals on the immune system, to address the concern that such compounds, by suppressing the immune response, may also make people more susceptible to infections and cancer. The authors of this review as well as several others were some of the early investigators who demonstrated how various chemicals used in the environment and industry could modulate the immune response, which also led to the establishment of immunological assays to screen for immunotoxicity [4,5,6,7,8].

Historically, it is exciting to know how the field of immunotoxicology, which began with the studies on TCDD and its receptor AhR leading to immunomodulation, also led more recently to characterization of natural and environmental ligands for AhR, and an understanding of how AhR activation triggers the regulation of T cell differentiation. Thus, within the span of 4 decades, the critical role of AhR is beginning to emerge, suggesting that it not only serves as an environmental sensor but also a key transcription factor that regulates several genes, especially those associated with the regulation of inflammation, leading to control of Regulatory T cells (Tregs) and inflammatory Th17 cells. It is very interesting to note that while the role of AhR in the field of toxicology was continuously developed though major advances in understanding how it regulated toxicity, its role in the field of immunology lagged behind in those 4 decades due to deficiencies in identifying the T cell subsets, especially the Tregs. Nonetheless, early studies on immunosuppressive effects of TCDD through activation of AhR set the stage for understanding the role of AhR in immunoregulation. Thus, this review highlights how the discovery of AhR in the field of immunotoxicology contributed towards better understanding of this transcription factor and its potential use in regulating inflammatory and autoimmune diseases.

## 2. TCDD Causes Toxicity and Immunosuppression through AhR: Early Studies

Since the discovery of AhR and its ligand TCDD, almost 45 years ago, there have been several studies trying to understand the nature of TCDD-induced toxicity in animals and humans [9,10,11,12,13,14,15,16,17,18,19,20,21]. Early reports suggested that TCDD not only caused overt toxicity in animals and but also caused immunosuppression [9,10,11,12,13,14,15,16,17,18,19,20,21]. Later, it was shown that TCDD acted through AhR present on a variety of immune cells [9,10,11,12,13,14,15,16,17,18,19,20,21].

As early as 1981, Clark et al. found that TCDD was very toxic, and, at a dose of 100 μg/kg or greater, produced cellular depletion in thymus, spleen, and lymph nodes in mice [22]. Moreover, the antibody responses to Sheep Red Blood Cells (SRBC) and TNP-*Brucella abortus*, as well as the delayed type hypersensitivity (DTH) response to oxazalone, were impaired following treatment with TCDD. TCDD caused a decrease in cytotoxic T lymphocyte (CTL) activity and in vitro analysis of the mechanism of suppression demonstrated that TCDD did not alter the precursor cells of CTL, but induced cells that could suppress CTL generation in vitro [22]. In 1984, one of the co-authors of this review, working in the Clark laboratory, reported that TCDD-induced susceptibility of mice to a variety of immunotoxic effects was genetically dictated by the presence of AhR locus [23]. In this report, it was shown that TCDD suppressed the generation of allo-specific CTL in the “susceptible” C57Bl/6 (Ah^b^), but not in “resistant” DBA-2(Ah^d^) [23]. To determine if TCDD acted directly on the “susceptible” lymphoid cells, the authors developed bone-marrow chimeras and found that the susceptibility to immunosuppression caused by TCDD in chimeric mice was influenced by the Ah genotype of the recipient host and not by the genotype of the donor’s grafted lymphomyeloid cells. Cell mixing experiments showed that the suppression of CTL generation by TCDD was caused by suppressor T cells [23]. These data were intriguing, and the recent observation that Tregs are relatively more radioresistant than other lymphocytes [24] may have led to this observation seen in chimeras.

Although early studies dating back to 1984 reported that TCDD caused the generation of “Suppressor T Cells” through the activation of AhR, it was difficult to identify these suppressor T cells due to a lack of specific cell markers. While some immunologists proposed that these suppressor cells express I-J molecules encoded by the Major Histocompatibility Complex (MHC), it was shown subsequently using molecular techniques that the I-J region did not exist in the MHC, which led to the rapid collapse of additional research on suppressor T cells [25]. Thus, unfortunately, further research that AhR activation by TCDD induces suppressor T cells was abandoned. It was not until 1995, when Sakaguchi et al. (1995) discovered T cells that suppressed immune response, which they called regulatory T cells (Tregs) [26], by identifying the expression of interleukin (IL)-2 receptor α-chain (CD25) on the cell surface of CD4^+^ T helper (Th) cell subtype, that the interest in T cells that could suppress immune response was once again ignited [26]. In 2001, T cells that suppress inflammation were further characterized with a specific biomarker, FoxP3, using scurfy mice that were devoid of this marker [27]. Bennet et al., in 2001, also reported that patients with the IPEX syndrome (immune dysregulation, polyendocrinopathy, enteropathy X-linked syndrome) carried mutations in the FoxP3, which led to dysfunctional FoxP3 protein expression and patients not developing functional Tregs, leading to lymphoproliferative and autoimmune disease [28]. A few years later, another breakthrough came in 2003, when FoxP3 was identified as the main transcription factor driving and maintaining Treg phenotype and function [29,30,31]. TCDD-induced Tregs/suppressor T cells received renewed attention with the discovery of a biomarker, FoxP3, as described in other reviews [32], which led to several novel studies on the mechanisms through which AhR activation by TCDD induced Tregs [33,34,35,36,37]. Around this time, there were also reports suggesting that TCDD may induce CD25^+^CD4^+^ Tregs that are FoxP3^−^ [38] or FoxP3^+^ [39], thereby suggesting that their induction may depend on the nature of the model and antigenic stimulation. Thus, identification of FoxP3 as a marker and master regulator of Tregs established the possible link between AhR and the regulation of inflammation and autoimmune disease. The focus of new studies was also to further understand the effect of TCDD on Treg subsets.

## 3. Tregs and Their Subsets

Studies revealed that Tregs belonged to two main subtypes: natural Tregs, which originate in the thymus (tTregs), and induced Tregs, which originate in the periphery (pTregs), following activation of naïve CD4^+^ T cells [25]. Although the term “regulatory T cell” was conventionally used to describe CD25^+^/FoxP3^+^ CD4^+^ T cells, it was shown that in the mouse, additional subtypes of Tregs could be demonstrated due to the lack or transient expression of FoxP3. For example, pTregs that produce high levels of IL-10 were classified as Tr1 (Treg type 1) cells, and Tregs producing high levels of TGF-β were classified as Th3 cells. Th3 cells can be FoxP3^−^ and shown to suppress autoimmune colitis [40,41]. Furthermore, it was noted that stable expression of FoxP3 was essential for Treg function and was sustained through epigenetic regulation of both in the *FoxP3* gene locus and Treg-specific demethylated region (TSDR) [42,43]. Naïve FoxP3^−^ CD4^+^ T cells in the mouse can express FoxP3 in the presence of transforming growth factor β (TGF-β) or retinoic acid, which promotes the development of peripherally induced Tregs.

## 4. AhR Ligands and How They Impact the Differentiation of Tregs vs. Th17

In recent years, there has been a significant surge in studies demonstrating that AhR activation by its ligands, natural or synthetic, triggers molecular mechanisms leading to ligand-specific regulation/differentiation of not only Tregs but also Th17 cells [33,35,36,37,39,44,45]. A list of AhR ligands and their potential effects on the immune response has been shown in Table 1. First, AhR was shown to be highly expressed by both Tregs and Th17 cells [37,45,46,47,48], while other T cell subsets such as Th1 and Th2 may express little or no AhR [49], thereby suggesting that AhR may primarily affect Th17/Treg differentiation. In 2008, Quintana et al. reported the identification of the ligand-activated AhR as a regulator of Treg and Th17 cell differentiation in mice [35]. They reported that AhR activation by 2,3,7,8-tetrachlorodibenzo-*p*-dioxin induced functional Tregs that suppressed experimental autoimmune encephalomyelitis (EAE) in mice [35]. AhR activation by 6-formylindolo(3,2-b)carbazole (FICZ), on the other hand, interfered with Treg’s development, boosted Th17 cell differentiation, and increased the severity of EAE in mice [35]. They proposed that AhR regulates both Treg and Th17 cell differentiation and the type of T cell differentiation may depend on the nature of AhR ligand. Around the same time, Veldhoen et al. also reported that AhR activation by FICZ was capable of stimulating myelin-reactive Th17 cells in an EAE model. More importantly, they found that CD4^+^ T cells from AhR-deficient mice could still develop into Th17 cells, but they lacked IL-22 expression. Their study concluded that stimulation of AhR by an environmental toxin promotes IL-22 production and enhances Th17 development [50]. Thus, when Veldhoen et al. studied EAE in AhR-deficient mice, they noted delayed onset and less severe disease compared to wildtype mice, correlating with a lack of functional Th17 cells in AhR-deficient mice [50]. In contrast, Quintana et al. used AhR^d^ mice with lower affinity binding of ligand to AhR, and found that these mice developed more severe forms of EAE than wildtype mice, which correlated with higher frequencies of Th17 cells [35]. The different responses seen in AhR-deficient mice versus those having AhR mutation (AhR^d^), which leads to decreased ligand affinity towards AhR, is intriguing and further suggests that ligand affinity or endogenous ligands present in AhR^d^ mice play a role in regulating neuroinflammation in this model.

Studies from our lab showed that TCDD-induced activation of AhR promoted the generation of Tregs that suppressed experimental colitis in mice [37]. In this study, we also observed that FICZ, on the other hand, promoted generation of Th17 cells, leading to enhancement of experimental colitis in mice [37]. In another study, we reported that indoles such as Indole-3-carbinol (I3C), which act as dietary AhR ligands, also promoted the generation of Tregs, suppressed differentiation of Th17 cells, and mitigated EAE in mice [45]. Additionally, we noted that dietary indoles promoted Tregs differentiation and suppressed Th17 cells differentiation, leading to suppression of Delayed-Type Hypersensitivity (DTH) in mice [48]. It was shown that dietary indoles suppressed DTH by inducing a switch from proinflammatory Th17 cells to anti-inflammatory Tregs through the regulation of miRNA [48]. All such studies clearly demonstrated that AhR activation by its ligands promoted Tregs/Th17 cells differentiation in a ligand specific manner [33,35,36,37,39,44,45].

Indoleamine-pyrrole 2,3-dioxygenase (IDO) is a heme-containing enzyme involved in tryptophan metabolism involving the kynurenine pathway. It is involved not only in tryptophan deprivation but also in the production of immunoactive kynurenines, which act as AhR ligands. Mature dendritic cells (DCs) that express IDO have been shown to induce proliferation of CD4^+^CD25^+^FoxP3^+^ Tregs [51]. In addition, IDO has been shown to promote the activation of Tregs while preventing the differentiation of Th-17 cells [52]. Interestingly, blocking IDO was shown to prevent Tregs and promote the differentiation of Th17 cells [53]. Such studies suggested that kynurenine produced by IDO, which acts as AhR ligand, may be responsible for promoting Tregs while inhibiting Th17 differentiation. DCs express AhR and in fact AhR also plays a critical role in the induction of IDO. Thus, AhR-deficient mice lack IDO and hence fail to produce Tregs while inducing Th-17 cells. However, addition of synthetic kynurenine to the T cell cultures polarized T cell differentiation more towards Tregs when compared to Th17 cells [54]. Together, these studies suggested that there is cross talk between AhR and IDO, and that IDO, through the induction of kynurenine, may also regulate Treg vs. Th-17 differentiation.

AhR ligands have also been shown to exert both oxidative and antioxidative properties [55,56,57]. Such ligands have been studied extensively in skin cells, such as keratinocytes, because they express significant levels of AhR [57]. Environmental toxicants such as TCDD and benzo[α]pyrene, primarily induce reactive oxygen species (ROS) in keratinocytes via AhR activation [56]. Such toxicants induce CYP1A1 and ROS generation, leading to DNA damage as well as interleukin 8 (IL-8) production in keratinocytes, which may account for their carcinogenic properties, and IL-8-driven inflammatory skin disorders such as psoriasis and palmoplantar pustulosis, respectively [57]. In contrast, several phytochemicals act as anti-oxidant ligands, including sulforaphane, soybean tar, cynaropicrin, *Opuntia ficus-indica* extract, and the like [55,56]. While these compounds activate AhR and induce CYP1A1 like TCDD, they inhibit ROS generation via the activation of nuclear factor-erythroid 2-related factor-2 (NRF2), an antioxidant master transcription factor [56]. Activation of NRF2 induces the transcription of a variety of antioxidant enzymes. The precise mechanisms through which environmental toxicants, versus phytochemicals, mediate differential effects, such as oxidative or antioxidative properties, following AhR activation, remain to be further elucidated. Oxidative stress can also affect T cell differentiation. For example, oxidative stress has also been shown to activate mTORC1, which in turn can activate inflammatory Th17 cells through the activation of STAT3 and ROR-gt [58,59]. In contrast, AhR ligands such as the phytochemicals that act as antioxidants may suppress inflammation by inducing Tregs and suppressing Th17 cells [60,61].

The T cell differentiation during AhR activation may also be driven by the cytokine and signaling pathways induced. There are studies showing that transforming growth factor (TGF)-β1 may induce the differentiation of Treg cells [62,63,64], while TGF-β1 along with combination of IL-6 or IL-21 induces the differentiation of Th17 [65,66,67,68]. Xingxing Liu et al. showed the role of STAT3 in AhR-regulated generation/suppression of Th17/Tregs [69]. They demonstrated that STAT3 might cooperate with AhR to regulate the differentiation of Th17 and Treg cells [69].

While the precise mechanisms that lead to such contrasting effects of AhR ligands on Treg/Th17 differentiation remain unclear, this may depend on the dose and duration of AhR activation [70], affinity of the ligand, and epigenetic alterations such as induction of miRNA that trigger pro- or anti-inflammatory activities [37,48,71], and the nature of microbiota [72]. In addition, because of the plasticity of T cells, and reciprocal regulation of Tregs vs. Th17 cells, it is possible that AhR activation may lead to the differentiation of either of these subsets based on the nature of antigenic stimulation, and cytokines that are found in the microenvironment. For example, it was shown that at early stages of Th17 cells differentiation, AhR activation might convert Th17 cells into IL-10-producing immunosuppressive Tr1 cells [73]. For the development of inflammatory Th17 cells, exposure to IL-23 is crucial. Thus, exposure to this cytokine during AhR activation of T cells may lead to inflammatory Th17 induction while its absence could lead to the generation of Tr1 cells [73].

Upon ligand binding, AhR can activate both the DRE-dependent and the DRE-independent protein–protein interaction pathway, which can regulate gene expression. Thus, in one study using three types of AHR ligands: agonist, antagonist, and selective AHR modulators (SAhRMs) that activate the non-DRE mediated pathway without activating the DRE-driven responses, it was shown that Th17 differentiation was mediated by the DRE-dependent pathway, and Treg differentiation was inhibited by suppressing the non-DRE pathway [74]. Another possibility of differential T cell response may result from the fact that TCDD has been shown to induce Activation Induced Cell Death (AICD) through apoptosis involving Fas–FasL activation [37,45,46,47,48]. Thus, this process may lead to the loss of antigen-activated T cells, leading to increased proportions of Tregs.

## 5. Epigenetic Regulation of T Cell Differentiation and Inflammation Following AhR Activation

Several recent studies from our lab have shown that both AhR-ligands such as TCDD and FICZ caused epigenetic modifications by regulating the expression of microRNAs (miRNAs), DNA methylation (hypomethylation and/or hypermethylation), and histone modification in the promoters of FoxP3 and IL-17 [37,45,48,71], which may regulate their differentiation. For example, dietary indoles such as I3C or 3,3′-diindolylmethane(DIM) attenuated delayed-type hypersensitivity (DTH) response to methylated Bovine Serum Albumin (BSA) through the suppression of Th17 cells while enhancing Tregs [48]. In contrast, FICZ increased the DTH response by enhancing Th17 cells. Indoles decreased the induction of IL-17 but promoted IL-10 and FoxP3 expression. microRNA (miR) analysis showed that I3C and DIM attenuated the expression of several miRs (miR-31, miR-219, and miR-490) that targeted FoxP3, while increasing the expression of miR-495 and miR-1192 that targeted IL-17 [48]. Surprisingly, FICZ caused opposite effects on these miRs. These studies demonstrated for the first time that the effect of AhR ligands to induce Tregs vs. Th17 cells may depend on the nature of miRs that they induce [48]. The role of miRs in inducing Tregs was also seen in another study using TCDD and pertussis-toxin mediated inflammatory response [71]. Some miRs may also regulate the expression of AhR and thereby influence the immune response. For example, miR124 was shown to play a critical role in the regulation of the inflammatory response of Chronic Rhinosinusitis (CRS) with nasal polyps through negatively regulating AhR expression [75].

TCDD was also shown to suppress colitis through the induction of Tregs while suppressing Th17 cells, and this was found to be associated with demethylation of CpG islands of FoxP3 and increased methylation in IL-17 promoter [37]. Recently, our lab demonstrated that TCDD caused significant induction of myeloid-derived suppressor cells (MDSCs) through AhR activation and miR regulation [76]. Because MDSCs are known to induce Tregs [77], it is likely that this may serve as yet another mechanism through which AhR activation triggers Treg differentiation. Alpinetin, a flavonoid, was found to suppress colitis through upregulation of miR-302 and decreasing the expression of DNMT-1, and thereby the methylation of FoxP3 promoter in CD4^+^ T cells in mice, leading to the induction of Tregs [78]. Deletion of AhR in T cells was shown to attenuate colitis, which results from suppressed Th17 cell infiltration into the lamina propria [79]. In this model, miRNA analysis showed that while colitis increased the expression of the miR-212/132 cluster, in AhR (−/−) mice, this expression was decreased. In addition, miR-212/132(−/−) mice were highly resistant to colitis, which correlated with decreased Th17 cells and increased IL-10-producing CD4^+^ cells [79], thereby establishing a connection between these miRs and Tr1 regulatory cells. Moreover, AhR activation leading to Th17 differentiation has also been shown to depend on the miR-132/212 cluster [80]. Dietary AhR ligands such as indoles (I3C and DIM) were shown to effectively attenuate Staphylococcal enterotoxin B (SEB)-mediated inflammation and liver injury by decreasing the expression of miR-31, which targeted the apoptotic pathways in activated T cells [81]. I3C may also act through the regulation of gut microbiota, as shown in a recent study in which AhR activation by I3C led to increased production of butyrate and attenuation of colitis. Thus, when mice with colitis were given butyrate, there was a decrease in colonic inflammation resulting from the suppression of Th17 and the induction of Tregs [72]. Additionally, IL-22 was increased following treatment with I3C but not with butyrate, and neutralization of IL-22 blocked the beneficial effects of I3C against colitis [72]. This study suggested that I3C activation of AhR attenuates colitis primarily through the induction of IL-22, which leads to dysbiosis that promotes the production of anti-inflammatory butyrate [72]. In addition, I3C and DIM have also been shown to decrease SEB-mediated inflammation by acting as HDAC-I inhibitors [82]. Below (Table 1) is the list of AhR ligands and their ability to induce Tregs or Th17 cells

**Table 1:** Natural and Synthetic AhR ligands and their effects on inflammation. It should be noted that some ligands may also act directly through other pathways such as NF-kB.

AhR Ligands	Origin Synthetic/Natural	Structure	Effect of AhR ligands on Inflammation and/or Tregs/Th17 Cells
2,3,7,8-Tetrachlorodibenzop- dioxin (TCDD)	Exogenous/Synthetic	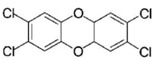	Promotes Tregs, suppresses Th17 cells, and attenuates inflammation [35,37]
Resveratrol	Dietary	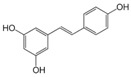	Promotes Tregs, suppresses Th17 cells, and attenuates inflammation [83,84]
lndole-3-carbinol (I3C)	Dietary	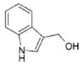	Promotes Tregs, suppresses Th17 cells, attenuates inflammation, and inhibits NF-kB [37,45]
3,3′-Diindolylmethane (DIM)	Dietary	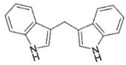	Promotes Tregs, suppresses Th17 cells, and attenuates inflammation [37,45]
Indolo[3,2-b]carbazole (ICZ)	Dietary	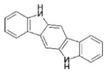	Attenuates inflammation [85,86]
lndole-3-acetonitrile (I3ACN)	Dietary	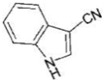	Anti-inflammatory and antioxidant [87]
2-(19H-indole-3′-carbonyl)- thiazole-4-carboxylic acid methyl ester (ITE)	Dietary	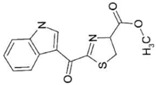	Promotes Tregs, suppresses Th17 cells, and attenuates inflammation [33,88]
2-(Indol-3-ylmethyl)-3,3′- diindolylmethane (Ltr-1)	Dietary	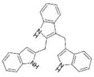	Attenuates inflammation [89,90]
Indole	Microbial	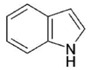	Promotes Tregs, suppresses Th17 cells, and attenuates inflammation [37,45]
lndole-3-acetic acid (IAA)	Microbial	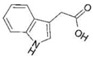	Anti-inflammatory and anti-oxidative [91,92]
lndole-3-aldehyde (IAld)	Microbial	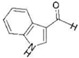	Attenuates inflammation [93]
Tryptamine	Microbial	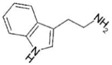	Attenuates inflammation [44,94]
3-Methyl-indole (skatole)	Microbial	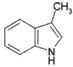	Attenuates inflammation [95,96]
Indirubin	Microbial/host metabolism/Plants	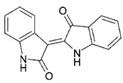	Promotes Tregs, suppresses Th17 cells, and attenuates inflammation [97,98,99]
lndoxyl-3-sulfate (I3S)	Microbial/host metabolism	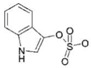	Attenuates inflammation [100,101,102]
Kynurenine (Kyn)	Host metabolism	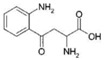	Promotes Tregs and attenuates inflammation [103]
Kynurenic acid (KA)	Host metabolism	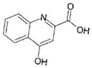	Promotes Tregs and attenuates inflammation [103]
Xanthurenic acid	Host metabolism	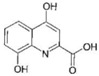	Anti-inflammatory [94]
Cinnabarinic acid (CA)	Host metabolism	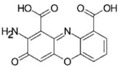	Attenuates inflammation [104,105]
6-Formylindolo[3,2-b]carbazole (FICZ)	Photo oxidation	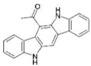	Suppresses Tregs, promotes Th17 cells, inflammation [35,37]

## 6. Conclusions

The aim of this review was to delineate how the current research involving the role of AhR in immunoregulation, specifically T cells, and its repercussion on the regulation of inflammatory and autoimmune diseases, originated from the field of immunotoxicology in the late 1970s and the early 1980s, when immunologists discovered that TCDD, an environmental pollutant, suppressed the immune response. While some studies clearly demonstrated in 1984 that TCDD triggered “suppressor T cells” [23], this observation did not receive further attention due to a lack of specific markers to identify these cells and distinguish them from other T cell subpopulations. Thus, the discovery of FoxP3 marker on CD4^+^ T cells, which led to the designation of the suppressor cells as Tregs, transformed the field, and immunologists began to unravel the mysteries of AhR not just as an environmental sensor involved in xenobiotic metabolism but also as a key regulator of immune functions.

**Figure 1:**
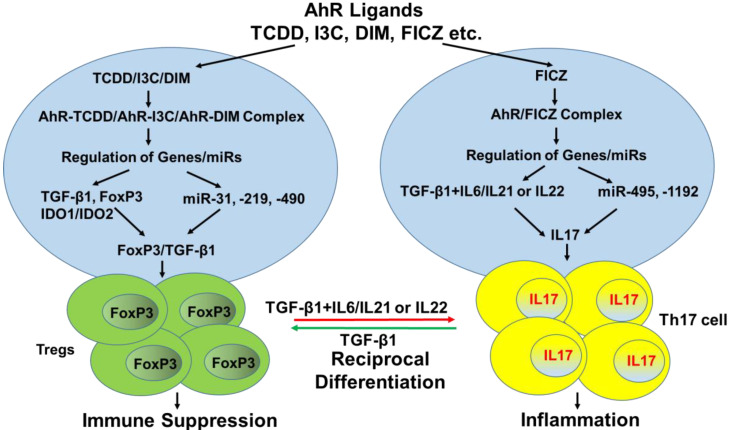
Signaling pathways that lead to the generation of Tregs versus Th17 cells following activation of AhR by a variety of ligands.

The field has advanced significantly in the past decade through the discovery of a variety of natural and synthetic ligands of AhR, which is helping us to understand how pro- and anti-inflammatory T cells are regulated. The differential regulation of Th17 vs. Treg differentiation by various AhR ligands (proposed in Figure 1) still constitutes an enigma and needs additional research. Activation of AhR also leads to epigenetic modulations in T cells, and the question of how such effects in turn regulate Treg vs. Th17 differentiation is exciting and clearly deserves additional investigation. The role of AhR in inflammatory and autoimmune diseases, particularly in colitis and MS, is receiving significant recognition, which means that there is substantial opportunity to target AhR using ligands to prevent and treat inflammatory and autoimmune diseases.
